# Epoxidized Vegetable Oils Plasticized Poly(lactic acid) Biocomposites: Mechanical, Thermal and Morphology Properties

**DOI:** 10.3390/molecules191016024

**Published:** 2014-10-08

**Authors:** Buong Woei Chieng, Nor Azowa Ibrahim, Yoon Yee Then, Yuet Ying Loo

**Affiliations:** 1 Department of Chemistry, Faculty of Science, Universiti Putra Malaysia, UPM Serdang 43400, Selangor, Malaysia; E-Mails: norazowa@upm.edu.my (N.A.I.); yoonyeetyy84@yahoo.com (Y.Y.T.); 2 Department of Food Science, Faculty of Food Science and Technology, Universiti Putra Malaysia, UPM Serdang 43400, Selangor, Malaysia; E-Mail: yuet_ying88@hotmail.com

**Keywords:** biocomposites, biodegradable, poly(lactic acid), epoxidized vegetable oils

## Abstract

Plasticized poly(lactic acid) PLA with epoxidized vegetable oils (EVO) were prepared using a melt blending method to improve the ductility of PLA. The plasticization of the PLA with EVO lowers the *T*_g_ as well as cold-crystallization temperature. The tensile properties demonstrated that the addition of EVO to PLA led to an increase of elongation at break, but a decrease of tensile modulus. Plasticized PLA showed improvement in the elongation at break by 2058% and 4060% with the addition of 5 wt % epoxidized palm oil (EPO) and mixture of epoxidized palm oil and soybean oil (EPSO), respectively. An increase in the tensile strength was also observed in the plasticized PLA with 1 wt % EPO and EPSO. The use of EVO increases the mobility of the polymeric chains, thereby improving the flexibility and plastic deformation of PLA. The SEM micrograph of the plasticized PLA showed good compatible morphologies without voids resulting from good interfacial adhesion between PLA and EVO. Based on the results of this study, EVO may be used as an environmentally friendly plasticizer that can improve the overall properties of PLA.

## 1. Introduction

Over the past few decades, most polymers used are petrochemically derived products that are non-biodegradable. The increase in global environmental problems such as green house gas emission and diminishing fossil resources has focused more attention on the development of green polymer composites. One such type of composites are bio-based polymer composites, which are environmentally friendly, compostable, biodegradable, and are acquired from renewable and sustainable resources [1]. They reduce our dependency on depleting fossil fuels and the generation of hazardous substances.

One of the most favorable materials for the production of high-performance, environmentally friendly biodegradable polymer is aliphatic polyester. Poly(lactic acid) (PLA), a linear aliphatic polyester, is regarded as most promising substitute for petroleum-based polymers due to its mechanical characteristics, such as tensile strength and Young’s modulus, similar to those of polyethylene terephthalate (PET) or nylon [2]. Moreover, PLA has good potential due to its excellent properties, such as high mechanical strength, transparency, compostability, moderate barrier capability and safety. Despite these desirable features, the high brittleness of PLA limits its application [1].

Therefore, considerable efforts have been made to enhance the characteristics of the polymer by employing plasticizer. Plasticizer is typically present in a range between about 1% to 10% by weight of polymeric material. Below 1%, the plasticizer may not effectively plasticizes the polymeric material, and above 10%, it tends to leach out of the polymeric material [3]. Petroleum based plasticizer are standard compounding ingredients, however epoxidized vegetable oil-based plasticizer is employed as a feasible alternative [4]. Vegetable oils are derived from plants and are chemically composed of different triacylglycerols,* i.e.*, esters of glycerol and fatty acids [5]. Vegetable oils are attractive raw materials for many industrial applications as they are derived from renewable resources, biodegradable, environmental friendly, easily available and produced in large quantities at a competitive cost [6]. Palm oil is a favorable vegetable oil because it is cheap, low in toxicity, and easily available as a sustainable agricultural resource. It comes from palm trees, one of the most economical perennial oil crops in Malaysia and belongs to the species *Elaeis guineensis* under the family *Palmacea*, originated in the tropical forests of West Africa. Epoxidized vegetable oils (EVO) are used extensively as plasticizers, stabilizers, and additives for many polymers [7]. Al-Mulla* et al.* studied the effects of epoxidized palm oil as plasticizer on the PLA/PCL blend prepared via the solution casting process [8]. The addition of epoxidized palm oil reduced the tensile strength and modulus but increased elongation at break for the PLA/PCL blend. The highest elongation at break was observed for the blend with 10 wt % epoxidized palm oil content. EVO are also a major raw material in the production of high functionality vegetable oil-based materials such as lubricants [9,10,11,12], alkyl nitrate triglyceride [13] and polyols [14,15].

In this study, two types of epoxidized vegetable oils (EVO), which are the epoxidized palm oil (EPO) and mixture of epoxidized palm oil and soybean oil (EPSO), are used as plasticizer to PLA via melt blending technique. The aim of this study was to investigate the effects of plasticizer loadings on the mechanical and thermal properties of PLA, as well as, to investigate the interaction between PLA and plasticizers. This material has great potential as alternatives to the conventionally used polymer such as polypropylene, as biodegradable or green biocomposites in the packaging industry.

## 2. Results and Discussion

### 2.1. Fourier Transform Infrared (FTIR) Spectroscopy

**Figure 1:**
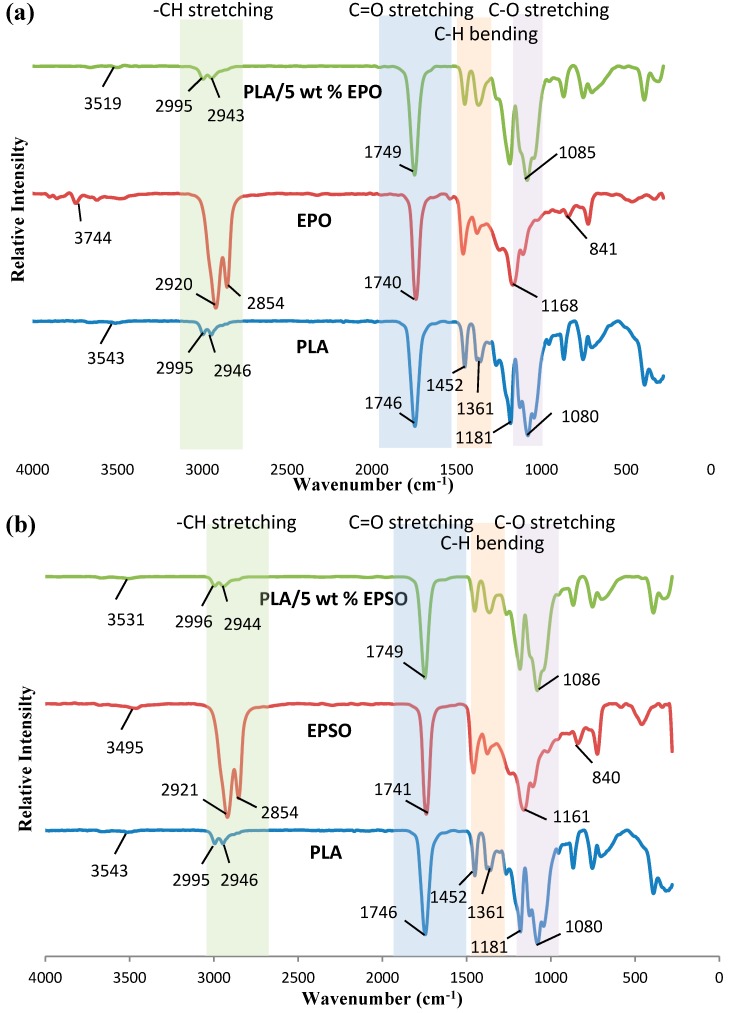
FTIR spectra of pristine PLA with (**a**) EPO and PLA/EPO; (**b**) EPSO and PLA/EPSO.

FTIR spectroscopy is used to monitor the absorption peak shift in specific regions to determine the known functional group interactions of the PLA with EVO. The FTIR spectra of PLA/EPO and PLA/EPSO are depicted in Figure 1a,b, respectively. The spectra show 4 main regions: -CH stretching at 3000–2850 cm^−1^, C=O stretching at 1750–1745 cm^−1^, C-H bending at 1500–1400 cm^−1^ and -C-O stretching at 1100–1000 cm^−1^. The FTIR spectra of EPO and EPSO exhibited the unique characteristic peaks that corresponded to the C-O-C stretching from oxirane vibrations at 950–850 cm^−1^ and around 1250 cm^−1^. The signal at 1250 cm^−1^ usually overlays with others, mainly -C-O which is present in oils.

For the plasticized PLAs, the peaks at about 3500 cm^−1^ indicates the presence of the free O–H stretching vibration from the production of EPO via acid catalyzed with hydrogen peroxide (H_2_O_2_) [16]. During the epoxidation with peracids, the acid produced simultaneously proceeds along with the reversible reaction with hydrogen peroxide to generate peracid again and free water group. A small amount of hydroxyl group (O–H) in the biocomposite could be attributed to the possible terminal hydroxyl groups in the PLA main chain which was released during the interaction between PLA and EPO [17].

**Figure 2:**
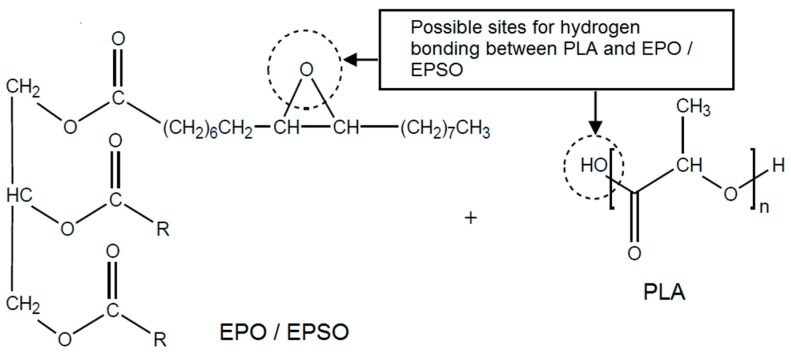
Proposed chemical interactions between PLA and Epoxidized Vegetable Oils.

A small shift of C-O stretching peak from 1080 cm^−1^ (neat PLA) to 1085 cm^−1^ in both PLA/EPO and PLA/EPSO were observed. This shift in the absorption peak indicates the miscibility and interaction of PLA and EVO. The upward shift may possibly due to an interaction between the hydroxyl group of PLA and the epoxy group of EVO through hydrogen bonding. A proposed possible interaction between PLA and EVO is shown in Figure 2.

The hydrogen bonding exists due the interaction between the polymer-plasticizer, PLA-EVO and is influenced by the epoxy content, also known as oxirane oxygen content (OOC) of the epoxidized oils. The OOC value shows the epoxy groups which exist in the plasticizer. It is essential for a good plasticizer to contain two types of structural components; the polar and non-polar components. The OOC represents the polar component other than the carbonyl group of carboxylic ester functionality. A higher OOC value of EPSO (3.58%) compared to EPO (3.23%) resembles stronger interaction between PLA and EPSO through hydrogen bonding compared to PLA and EPO. According to George Wypych, polar groups in a plasticizer improve mechanical properties and are essential for good compatibility [18]. Eventually, if the plasticizer used is very non-polar (low OOC value but high iodine value), it results in poor interaction and eventually less compatibility between polymer and plasticizer, as found with PLA/EPO, which leads to lower mechanical properties compared to PLA/EPSO.

### 2.2. Mechanical Properties

**Figure 3:**
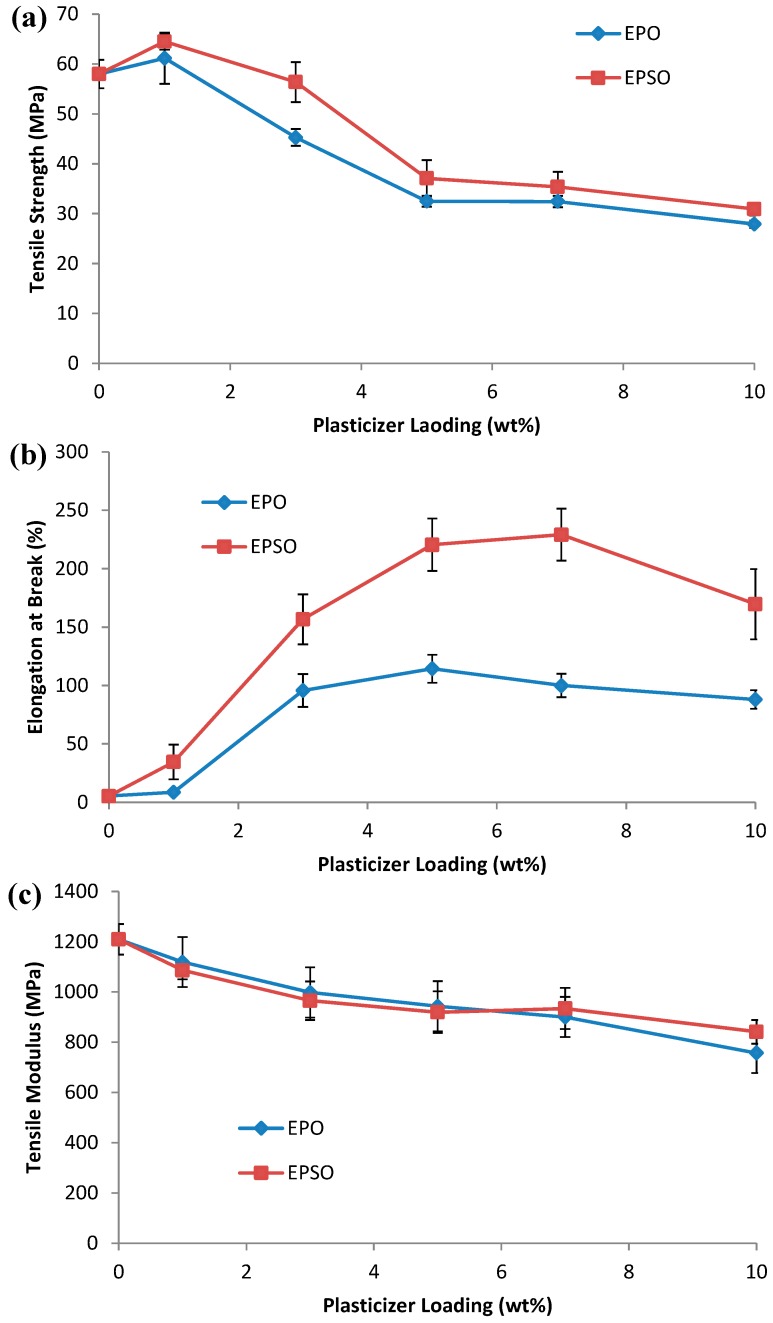
(**a**) Tensile strength; (**b**) elongation at break and (**c**) tensile modulus of plasticized PLA.

Tensile properties are the most frequently used indicator of change caused by plasticization. The addition of 1 wt % EVO plasticizers into PLA matrix significantly improves the tensile strength of PLA. PLA/1 wt % EPO and PLA/1 wt % EPSO show increments of approximately 5% and 11%, respectively as shown in Figure 3a, compared to neat PLA. The tensile strength of PLA decreases with the addition of plasticizers above 1 wt %. The drop in tensile strength may be caused by the formation of plasticizer-plasticizer interaction which dominates at higher EPO or EPSO contents, resulting in a phase separated structure. In addition, at higher plasticizer loading, only a part of the plasticizer was located in the interfacial area, while the remaining is spread in the matrix, influencing the homogeneity and causing the drop in the tensile strength of the plasticized PLA [19].

The elongation at break of PLA increases with the addition of plasticizers. Neat PLA exhibits an elongation at break of 5.3%. It has been observed in Figure 3b that elongation at break of PLA increases significantly with increasing plasticizers (EPO and EPSO) loading until an optimum point. Addition after this point causes the decrease of elongation at break, making the biocomposite more brittle. With 5 wt % plasticizers loadings, PLA/EPO and PLA/EPSO displayed elongation at break of 114.4%, and 220.5% respectively. In general, plasticizer is introduced to a polymer matrix to overcome the brittleness caused by extensive intermolecular interactions. Thus, the presence of plasticizers EPO and EPSO decreases these intermolecular forces and enhances the mobility of PLA polymer chains, causing an increase in flexibility and extensibility of the PLA. Several theories have been proposed to explain the mechanism and action of plasticizers on polymers. Among those theories, lubricity theory and gel theory have been widely accepted to describe the effect of plasticizers on polymeric networks. Lubricity theory mentions that the plasticizer acts as a lubricant to reduce friction and facilitates polymer chain mobility past one another, consequently lowering deformation. Gel theory extends the lubricity theory and suggests that a plasticizer disrupts and replaces polymer–polymer interactions (hydrogen bonds, van der Waals or ionic forces, *etc.*) that hold polymer chains together resulting in reduction of the polymer gel structure and increased flexibility.

Since the plasticizer plasticizes polymers, the typical expectation is that the tensile modulus of plasticized material decreases with increased amounts of plasticizer as shown in Figure 3c. The PLA exhibited a tensile modulus value of 1209 MPa. The addition of 10 wt % EPO and 10 wt % EPSO reduces the stiffness of PLA to 757 MPa and 841 MPa, respectively. This was attributed to the toughening and elastomeric effect of EPO and EPSO. EPO and EPSO, which contain the epoxy group, could form favorable interactions with PLA, presumably via hydrogen bonding as proposed in Figure 2.

It should be noted that the tensile strength and elongation at break in PLA/EPSO is slightly higher compared to PLA/EPO. This is due to the interaction between polymer and plasticizer, which can be explained by the epoxy content also known as oxirane oxygen content (OCC) of the epoxidized oil. The OCC value indicates the epoxy groups present in the plasticizer. Higher OCC value in EPSO (3.58%) compared to EPO (3.23%) resembles stronger interaction (hydrogen bonding) between PLA and EPSO, which gives better tensile properties. Based on the result of elongation at break of plasticized PLAs, the PLA plasticized with 5 wt % EPO, 5 wt % EPSO showed the best results and were selected for further study and compared to pristine PLA. 

### 2.3. Dynamic Mechanical Analysis (DMA)

Dynamic mechanical analysis (DMA) is a method in which the elastic and viscous response of a sample under oscillating load, are monitored as a function of temperature. DMA results are expressed by three parameters: the storage modulus (E'), the loss modulus (E''), and the tan δ (E''/E' ratio). During a heating at constant frequency, the storage modulus (E'), usually strongly decreases when temperature crosses the dynamic glass transition. On the other hand, the loss modulus (E''), and the loss factor (tan δ) exhibit a peaked shape.

PLA is a semicrystalline material, and its storage modulus begins to decrease rapidly at 50 °C as the material enters its glass transition. Because of its crystalline properties, it displays a region of relative stability before its modulus plummets rapidly as its structure approaches the melting point.

**Figure 4:**
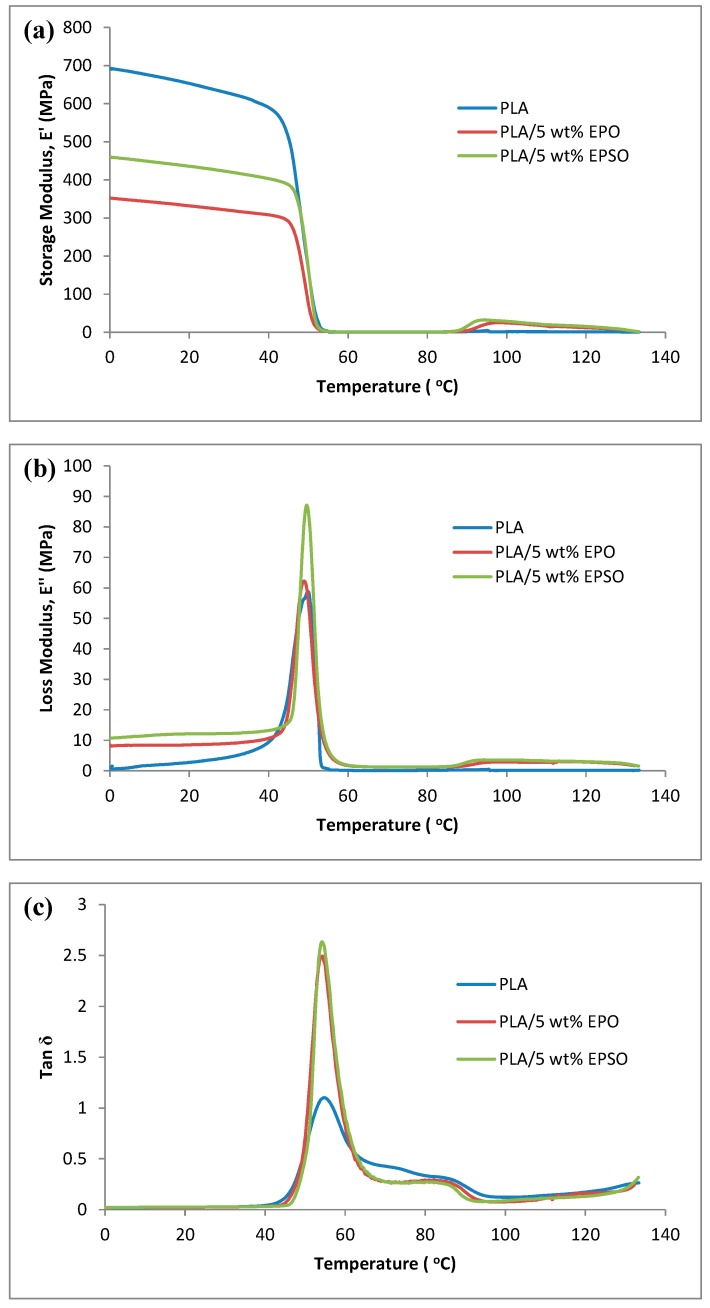
(**a**) Storage modulus; (**b**) loss modulus and (**c**) tan δ of PLA and plasticized PLAs as a function of temperature.

The DMA analysis for the plasticized PLA was carried out to see the effect of the plasticizers on the thermomechanical properties. Figure 4a–c illustrates the dynamic storage modulus, loss modulus and tan δ of PLA and plasticized PLAs, as a function of temperature, respectively.

The lower storage modulus of the plasticized PLA compared to neat PLA can be seen in Figure 4a indicating an increase in the flexibility of PLA imparted by the plasticizers. The storage modulus of the plasticized PLAs decreased with the presence of plasticizer below 50 °C. Further, there was a large drop on the storage modulus around 50–60 °C corresponding to the glass transition region. The storage modulus of the plasticized PLAs decreased, indicating that plasticizers increase the mobility of the PLA molecular chain. The storage modulus value measurements were in agreement with the measurements of tensile modulus values from tensile test. Besides, the plasticizer provides moderate toughening and elastomeric effect, which brings about a decrease in the modulus values. Reduction in storage modulus with respect to temperature is related to softening of the matrix at higher temperature. As the temperature exceeds the softening point, the mobility of the matrix chains increases, leading to the sharp decrease of modulus at temperatures between 50–60 °C [20].

Figure 4b shows the loss modulus of PLA and plasticized PLAs. The peak intensity of loss modulus curve signifies the melt viscosity of a polymer. As observed, the enhancement of loss modulus is higher in plasticized PLAs compared to neat PLA. This indicates that the incorporation of plasticizer increases the melt viscosity of PLA by acting as a good solvent or plasticizer [21]. This is attributable to the addition of EVO which increases the flowability by triggering the PLA polymer chains to align in the direction of flow, owing to a less rigid polymeric material [22]. PLA/EPSO shows higher melt viscosity due to improved interphase interaction between PLA and EPSO through hydrogen bonding, compared to PLA/EPO, and have higher ability to dissipate mechanical energy through molecular motion.

The tan δ peak has been used to investigate the glass transition of semicrystalline polymer or polymeric networks. The temperature dependence of tan δ of PLA and plasticized PLAs are presented in Figure 4c. The tan δ curves show two dynamic relaxation peaks at 80–90 °C and 50–60 °C, which are referred to as α and β-relaxation peaks, respectively. It is assumed that the β-relaxation peak is linked to the breakage of the hydrogen bonding between polymer chains, inducing long-range segmental chain movement in the PLA matrix. Therefore, the β-relaxation peak at 50–60 °C was assigned to the glass transition temperature, *T*_g_. The *T*_g_ of PLA is about 55 °C and the plasticized PLAs have slight shifts to lower *T*_g_ temperature, as shown in the Figure 4c. The glass transition temperatures were decreased slightly as a result of the plasticization effect. 

On the other hand, it is observed from Figure 4c that there is a slight increase in the intensity of the β-relaxation peak. Since the glass transition process is linked to the molecular motion, the *T*_g_ is considered to be influenced by molecular packing, chain rigidity and linearity as well. Since the intensity of the β-relaxation peak is associated to molecular mobility, it has been observed that the incorporation of plasticizers into the PLA polymer matrix increases their molecular mobility and in turn increases the intensity of the relaxation peak.

### 2.4. Thermal Properties

#### 2.4.1. Thermogravimetry Analysis (TGA)

**Figure 5:**
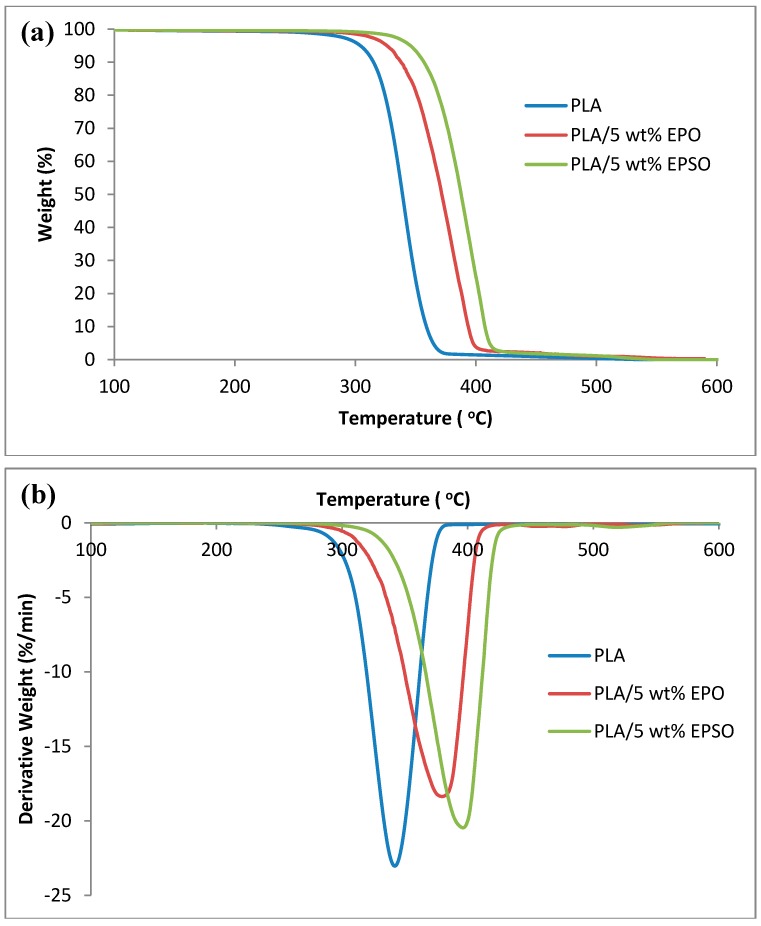
Effect of plasticizers on thermal stability of PLA (**a**) TGA; (**b**) DTG.

The thermal degradation behavior of the plasticized PLAs was studied with TGA. The TGA thermograms are shown in Figure 5. Thermal stability factors, including initial decomposition temperature (*T*_onset_), temperature of maximum rate of degradation (*T*_max_) and decomposition temperature at 50% weight loss (*T*_50_) of the plasticized PLA, can be determined from the TGA thermograms. As observed in Figure 5, the decomposition curve behavior of the plasticized PLAs is largely similar to those of neat PLA and takes place in a single weight loss step.

The *T*_onset_, *T*_max_ and *T*_50_ of the plasticized PLAs are tabulated in Table 1. It can be seen that the decomposition temperature of the plasticized PLAs commences near 300 °C and rapidly continues until 430 °C. The degradation onset temperatures of PLA/EPO and PLA/EPSO are higher than that of neat PLA. Neat PLA has an onset temperature of 274.26 °C, which is increased to 313.54 °C and 330.40 °C when 5 wt % of EPO and EPSO, respectively, are incorporated into PLA. From the results, it can be confirmed that the plasticized PLAs containing 5 wt % plasticizer show excellent thermal stabilities, which can be attributed to the excellent network structure in the biocomposites. For example, neat PLA has *T*_max_ of 345.12 °C, which is increased to 379.79 °C and 396.34 °C when EPO and EPSO, respectively, are incorporated into the PLA.

**Table 1:** Thermal degradation temperatures of PLA and plasticized PLAs.

	*T*_onset_ (°C)	*T*_max_ (°C)	*T*_50_ (°C)
PLA	274.26	345.12	339.16
PLA/5 wt % EPO	313.54	379.79	371.67
PLA/5 wt % EPSO	330.40	396.34	387.77

#### 2.4.2. Differential Scanning Calorimetry (DSC)

Differential Scanning Calorimetry (DSC) measures the amount of heat energy absorbed or released when the material is heated or cooled. For polymeric materials, which undergo important property changes near thermal transition, DSC is a very useful technique to study the glass transition temperature, crystallization temperature, and melting behavior.

Pristine PLA showed an endothermic peak of melting, *T*_m_ = 149.79 °C. A minor decrease in the melting temperature of 3 to 4 °C was observed, indicating that the melting temperature of PLA was not greatly affected by the addition of EVO plasticizer.

**Figure 6:**
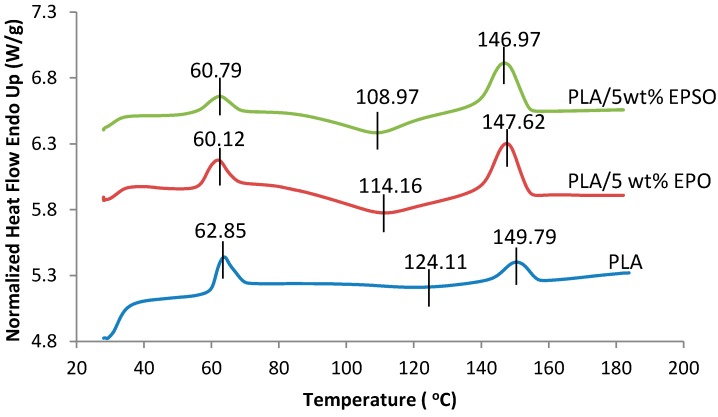
DSC thermograms of PLA and plasticized PLAs.

The pristine PLA showed a sharp *T*_g_ and its value decreased gradually with addition of EVO as shown in Figure 6. *T*_g_ decreased from 62.85 °C for pristine PLA to 60.12 °C and 60.79 °C when 5 wt % of EPO and EPSO respectively was added due to enhanced segmental mobility of PLA chains caused by the presence of EVO plasticizers. No trace of separate melting or crystallization of EVO was found confirming that the phase separation of EVO did not occur.

Cold-crystallization was chosen as a crystallization method because it leads to a more intense spherulite nucleation resulting in shorter crystallization time and smaller spherulite sizes [23]. Pristine PLA showed cold-crystallization temperature at about 124.11 °C. The cold-crystallization temperature of PLA decreased with EVO addition, in parallel with the shift in *T*_g_ as shown in Figure 6. The cold-crystallization decreased to 114.16 °C and 108.97 °C for the biocomposite containing 5 wt % EPO and EPSO, respectively. The depression of *T*_cc_ and the decrease in *T*_g_ indicated that the EVO was compatible with PLA [1]. Enhanced chain mobility increased the rate of crystallization, which allowed PLA to crystallize at lower temperature. Furthermore, the crystallization peak of the biocomposites was narrowed due to increased ability of PLA to crystallize [24].

### 2.5. Scanning Electron Microscopy (SEM)

**Figure 7:**
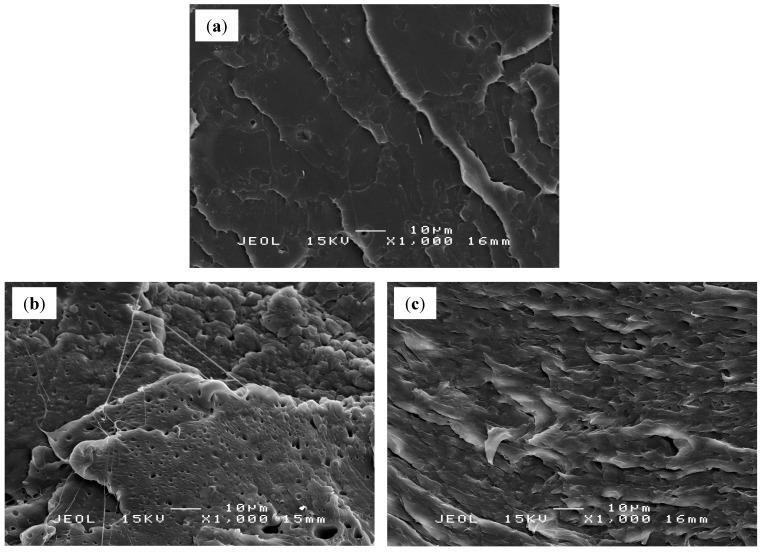
SEM micrographs of (**a**) PLA, (**b**) PLA/EPO and (**c**) PLA/EPSO.

Scanning electron microscopy (SEM) was employed to discern the surface morphology of the fractured tensile specimens and qualitatively illustrate the state of dispersion of the EVO in the polymer matrix. A typical fracture surface of PLA is shown in Figure 7a, which exhibited a flat surface corresponding to brittle crack growth behavior [19]. The addition of EVO as plasticizer to PLA matrix determined a marked change in the morphology with improved interfacial adhesion and dispersion. 

SEM micrographs of PLA/5 wt % EPSO, Figure 7c, show very good compatible morphologies without edge, cavity, and holes compared to PLA/5 wt % EPO (Figure 7b). This incidentally means more transfer of loads under stress conditions, consistent with the enhanced tensile properties of the biocomposites. This phenomenon shows a good adhesion between the components with a diffused polymer-plasticizer interface, which is attributed to the occurrence of chemical interactions between PLA and EVO. Thus, the EVO was well-dispersed to form a homogeneous matrix with evident signs of plasticization in the PLA matrix, without separation at the interface producing single phase morphology [25].

## 3. Experimental Section

### 3.1. Materials

Poly(lactic acid) resin, commercial grade 4042D, Mw ~ 390,000 Da, was supplied by NatureWorks^®^ LCC, Minnetonka, MN, USA. Epoxidized palm oil (EPO) and mixture of epoxidized palm oil and soybean oil (EPSO) supplied by Advanced Oleochemical Technology Division (AOTD), Malaysian Palm Oil Board (MPOB, Kajang, Malaysia). The characteristics of the EPO obtained are listed in Table 2.

**Table 2:** Properties of EPO and EPSO.

Sample Composition	Epoxidized Palm Oil (EPO)	Mixture of Epoxidized Palm Oil and Soybean Oil (EPSO)
Oxygen Oxirane Content (%)	3.2309	3.5803
Acid Value (mg KOH/g sample)	0.4287	0.5990
Iodine Value (g I_2_/100 g sample)	0.6371	0.4999
Moisture Content	0.08	0.06
pH	5–6	5–6

### 3.2. Preparation of PLA/EVO Biocomposites

The PLA/EVO biocomposites were prepared by melt blending technique using Brabender Internal Mixer at 170 °C with 50 rpm of the rotor speed. The plasticizer was added after 2 min of blending PLA and continues for another 8 min. The weight of EVO studied was varied from 0 to 10 wt %. The biocomposites obtained were then molded into sheets of 1 mm in thickness by hot pressing at 165 °C for 10 min with pressure of 110 kg/cm^2^, followed by cooling to room temperature. The sheets were used for further characterization. 

### 3.3. Characterizations

#### 3.3.1. Fourier Transform Infrared (FTIR) Spectra

The FTIR spectra of biocomposites and raw materials were recorded using a Fourier Transform Infrared Spectrometer (Perkin-Elmer: Model 1000 Series) instrument equipped with a universal attenuated total reflectance (UATR) accessory. The spectra were recorded between 4000 cm^−1^ and 280 cm^−1^ frequency ranges. The data were analyzed using the program FTIR Spectrum Software (Perkin Elmer).

#### 3.3.2. Tensile Properties Measurement

Tensile properties were tested using Instron 4302 series IX (Buckinghamshire, UK). The samples were cut into dumbbell shapes following ASTM D638 (type V) standard. A load of 1.0 kN was applied at constant crosshead speed of 10 mm/min at room temperature. The Tensile strength, tensile modulus and elongation at break were evaluated from the stress-strain data. Each sample included seven tested replicates to obtain a reliable mean and standard deviation.

#### 3.3.3. Dynamic Mechanical Analysis

Thermal dynamic analysis (DMA) was performed according to ASTM D5023 on a dynamic mechanical analyzer (Perkin-Elmer PYRIS Diamond DMA), using bending mode. The temperature scan was from the ambient temperature (25 °C) to 150 °C at a constant heating rate of 2 °C/min and the frequency of dynamic force of 1 Hz, under nitrogen atmosphere. The storage modulus (E'), loss modulus (E''), loss factor (tan δ), and glass transition temperature of each specimen were obtained as a function of temperature.

#### 3.3.4. Thermal Properties

Differential Scanning Calorimetry (DSC) analysis was performed by Perkin Elmer JADE DSC to study the nonisothermal crystallization kinetics. The DCS procedure consisted of three steps. At the first step, the films were heated from 30 to 180 °C with a heating rate of 10 °C/min. Then, they were held at this temperature for 5 min to eliminate the thermal history, and they were cooled to 30 °C at a cooling rate of 10 °C/min and held at 30 °C for 5 min. In the last step, they were reheated to 180 °C at a heating rate of 10 °C/min.

Thermogravimetric analysis (TGA) was carried out using a Perkin Elmer Pyris 7 TGA analyzer with scan range from 35° to 800° at a constant heating rate of 10 °C/min and continuous nitrogen flow. The thermal degradation temperatures taken into account were the temperature at onset (*T*_onset_), the temperature of maximum weight loss (*T*_max_) and temperature at 50% weight loss (*T*_50_).

#### 3.3.5. Morphology

The fracture surfaces of tensile failed sample were studied under a JEOL scanning electron microscopy (SEM) instrument JSM-6400 (Tokyo, Japan) at an accelerating voltage of 30 kV. The fractured surfaces were coated with a thin layer of gold prior to observation. 

## 4. Conclusions

PLA plasticized with different epoxidized vegetable oils (EVO) have been successfully prepared. Tensile test showed that the optimum improvement in the mechanical properties was achieved when 5 wt % of EVO was introduced into the PLA matrix. Furthermore, SEM analysis revealed better miscibility and interfacial adhesion between PLA and 5 wt % EVO. Consequently, the main goal to improve the flexibility of PLA was achieved. These findings support that the EVO can be used as an excellent plasticizer, which increases the interaction at the phase boundaries and overall properties. Further, PLA/EVO biocomposites can be used as biodegradable or green composite alternatives to the conventionally used polymers, such as polypropylene. 
